# Frailty Impact on In-Hospital Outcomes Among Patients Hospitalized with Acute Pulmonary Embolism: A National Inpatient Sample Analysis

**DOI:** 10.3390/jcm15208107

**Published:** 2026-10-19

**Authors:** Daniel Carny, Adam Folman, Rami Abu Fana, Maguli Barel, Ariel Roguin, Ofer Kobo

**Affiliations:** 1 Rambam Health Care Campus, Haifa 3109601, Israel; daniel.carny10@gmail.com; 2 Ruth and Bruce Rappaport Faculty of Medicine, Technion—Israel Institute of Technology, Haifa 3200003, Israel; 3 Division of Cardiovascular Medicine, Hillel Yaffe Medical Center, Hadera 38100, Israel

**Keywords:** pulmonary embolism, frailty, hospital frailty risk score, in-hospital mortality, risk stratification, national inpatient sample, healthcare utilization

## Abstract

**Background/Objectives**: Acute pulmonary embolism (PE) can be life-threatening, and outcomes vary considerably between patients. Frailty reflects reduced physiologic reserve and may help describe vulnerability during an acute illness. We examined the association between the Hospital Frailty Risk Score (HFRS) and in-hospital outcomes among adults admitted with acute PE. **Methods**: We analyzed adult hospitalizations for acute PE recorded in the U.S. National Inpatient Sample between 2016 and 2021. HFRS was reconstructed from 109 three-character ICD-10 categories, with each category counted once per hospitalization and grouped as low (<5), intermediate (5–15), or high (>15). Survey-weighted logistic regression used NIS discharge weights, year-specific strata, and year-specific hospital clusters. Additional analyses were limited to patients aged ≥65 and ≥75 years, adjusted for available PE-severity markers and repeated with a modified HFRS that excluded potentially acute components. **Results**: The study included 220,455 unweighted hospitalizations, representing an estimated 1,102,274 U.S. hospitalizations. Low, intermediate, and high HFRS categories accounted for 67.1%, 31.5%, and 1.5%, respectively. In-hospital mortality was 1.2%, 7.1%, and 9.7% across these groups. Mechanical ventilation occurred in 0.9%, 7.9%, and 16.2%, acute ischemic stroke in 0.2%, 1.3%, and 6.4%, and major bleeding in 1.2%, 4.8%, and 9.3%, respectively. Compared with low HFRS, high HFRS was associated with higher odds of mortality (aOR 8.30, 95% CI 7.24–9.52), mechanical ventilation (aOR 27.74, 95% CI 24.59–31.31), circulatory support (aOR 19.18, 95% CI 11.83–31.11), acute ischemic stroke (aOR 37.61, 95% CI 30.45–46.46), and major bleeding (aOR 7.65, 95% CI 6.68–8.76). Associations persisted in age-restricted and severity-adjusted analyses but were substantially attenuated when potentially acute HFRS components were excluded. **Conclusions**: Higher HFRS categories were associated with worse in-hospital outcomes in acute PE. Associations were weaker after removing HFRS components that may reflect acute conditions, suggesting that same-admission coding may explain part of the association. Further prospective studies are needed before HFRS is used for treatment decisions or as an additional prognostic tool in acute PE.

## 1. Introduction

Acute pulmonary embolism (PE) is a common and potentially fatal cardiovascular condition [1]. Among cardiovascular causes of death, PE ranks behind myocardial infarction and stroke and contributes substantially to healthcare use worldwide [1,2,3]. Diagnostic and treatment options have expanded, including anticoagulation, systemic thrombolysis, and catheter-based therapy, but outcomes still differ considerably between patients [4].

Early risk assessment is an important part of PE management. Current approaches use hemodynamic findings, imaging, and biomarkers to estimate short-term severity [5]. These measures mainly describe the acute event and may not fully reflect how well an individual patient can tolerate it. Functional status, chronic disease burden, and physiologic reserve can differ substantially even among patients with similar PE severity [6].

Frailty is a multidimensional syndrome marked by reduced physiologic reserve and a limited ability to respond to acute stress [7,8]. In cardiovascular populations, frailty has been associated with higher mortality, more complications, longer hospital stays, and greater healthcare use [9,10,11]. Frailty is not fully captured by chronological age or standard comorbidity measures [12].

The Hospital Frailty Risk Score (HFRS) is an administrative-data measure developed to identify older hospitalized patients at increased risk of adverse outcomes [13,14]. It is based on 109 three-character ICD-10 diagnosis categories, each treated as a binary indicator and assigned a predefined weight. The original score was developed in patients aged 75 years or older and can incorporate diagnoses from the index admission and prior emergency admissions. Because the NIS is discharge-based and does not permit longitudinal linkage of individual patients, only diagnoses recorded during the index hospitalization were available in the present study. This difference is important when interpreting the score in this cohort.

Previous studies suggest that frailty is associated with worse outcomes in patients with acute PE, including higher short-term mortality and longer hospital stay [15,16]. However, most studies have been single-center, have used a binary definition of frailty, or have not examined individual in-hospital complications and discharge disposition in detail. The association between frailty severity and these outcomes at the national level therefore remains uncertain.

In the present study, we used a large nationally representative cohort of patients hospitalized with acute PE to examine the association between HFRS category and in-hospital outcomes. We also performed sensitivity analyses addressing age, available PE-severity markers, comorbidity burden, and possible overlap between acute diagnoses and HFRS components.

## 2. Materials and Methods

### 2.1. Data Source

We analyzed the 2016–2021 National Inpatient Sample (NIS). This Healthcare Cost and Utilization Project (HCUP) database contains a stratified sample of U.S. inpatient discharges from all payer types and represents about one fifth of national hospital discharges. Available fields include patient and hospital characteristics, diagnosis and procedure codes, length of stay, hospital charges, and discharge status. HCUP discharge weights were used to obtain national estimates.

### 2.2. Cohort Selection

The study population was limited to adults aged ≥18 years whose principal discharge diagnosis was acute PE, identified with ICD-10-CM codes. Records carrying only chronic or prior-history PE codes were not included. After these criteria were applied, 220,455 sampled discharges remained, corresponding to 1,102,274 weighted hospitalizations during 2016–2021.

### 2.3. Frailty Assessment

Frailty was quantified with the Hospital Frailty Risk Score (HFRS), using the 109 three-character ICD-10 categories and weights described by Gilbert et al. [13]. Each category was coded as present or absent and could contribute its weight only once per hospitalization. The weighted values were summed to obtain the total score, categorized as low (<5), intermediate (5–15, inclusive), or high (>15). The NIS does not provide diagnosis-level present-on-admission indicators, and patients cannot be linked across admissions. HFRS was therefore based only on diagnoses recorded during the index hospitalization. Some acute conditions that developed during admission may therefore have contributed to the score; this was examined in sensitivity analyses.

### 2.4. Baseline Variables

Patient and hospital characteristics included age, sex, race, weekend admission, payer, household income by ZIP code, hospital region, teaching status, and hospital bed size. Chronic comorbidities were identified using ICD-10-CM diagnosis codes. Acute in-hospital events were not presented as baseline comorbidities in Table 1.

### 2.5. Outcomes

The main in-hospital outcomes were all-cause in-hospital mortality, mechanical ventilation, circulatory support, acute ischemic stroke, and major bleeding. Diagnosis- and procedure-based outcomes were reconstructed from the raw ICD-10-CM/PCS fields using prespecified code sets. Major bleeding was defined as intracranial hemorrhage, gastrointestinal bleeding, or retroperitoneal bleeding. Secondary outcomes were hospital length of stay, total hospital charges, and discharge disposition. Exact outcome definitions are provided in Supplementary Table S4.

### 2.6. Statistical Analysis

Descriptive analyses accounted for the NIS survey design. Continuous variables are reported as means or, when appropriate, medians with interquartile ranges; categorical variables are shown as weighted percentages. The *p* values in Table 1 and Table 2 compare the three HFRS categories overall and should not be interpreted as pairwise comparisons or linear-trend tests.

For each binary outcome, we fit a survey-weighted logistic regression model with HFRS category as the exposure and the low-HFRS group as the reference.

The primary adjusted model included age, sex, race, primary payer, ZIP-code income quartile, weekend admission, hospital region, hospital location/teaching status, bed size, hyperlipidemia, valvular heart disease, prior myocardial infarction, obesity, smoking status, and malignancy. Conditions that form part of the HFRS were not added again to the primary model.

Because diagnosis timing is not available in the NIS, acute events were not included in the primary adjustment model. To examine potential confounding by PE severity, a sensitivity model additionally adjusted for saddle PE, cardiogenic shock, cardiac arrest, ventricular tachycardia, and ventricular fibrillation. A separate broader diagnosis-coded comorbidity model additionally included diabetes mellitus, dementia, chronic kidney disease, hypertension, heart failure, atrial fibrillation, chronic lung disease, anemia, thrombocytopenia, coagulopathy, peripheral vascular disease, and chronic liver disease.

Adjusted odds ratios (aORs) with 95% confidence intervals (CIs) were reported. Survey-weighted models used the NIS discharge weight (DISCWT). Variance estimation used Taylor linearization with year-specific NIS strata and year-specific hospital identifiers as primary sampling units; singleton strata were treated as certainty strata. Additional analyses were restricted to patients aged ≥65 and ≥75 years. To examine possible overlap between acute diagnoses and HFRS, we recalculated HFRS after excluding 19 categories that may reflect acute complications (N39, F05, B96, R56, R40, S06, E87, E86, R55, N17, B95, A41, I95, J96, I63, J18, J22, Y95, and R50). Because very few hospitalizations remained in the modified high-HFRS category, this analysis compared modified HFRS ≥5 versus <5. For in-hospital mortality, we also compared weighted C-statistics and Brier scores for models with and without HFRS. Analyses were performed in Python 3.13.5 (Python Software Foundation, Wilmington, DE, USA) using pandas 2.2.3, statsmodels 0.14.6, NumPy 2.3.5, and SciPy 1.17.0. A two-sided *p* value < 0.05 was considered statistically significant.

## 3. Results

### 3.1. Patient Characteristics

The cohort included 220,455 unweighted hospitalizations, corresponding to an estimated 1,102,274 hospitalizations after application of NIS discharge weights. Of these, 67.1% were classified as low HFRS, 31.5% as intermediate HFRS, and 1.5% as high HFRS. Patient and hospital characteristics are shown in Table 1.

Patients in higher HFRS categories were older and had a greater burden of several chronic conditions. Mean age was 60.1 years in the low-HFRS group, 69.0 years in the intermediate group, and 75.0 years in the high group. Women accounted for 50.0%, 55.2%, and 58.4% of the three groups, respectively. Dementia was present in 1.5% of the low-HFRS group, 13.9% of the intermediate group, and 39.2% of the high group; chronic kidney disease was present in 7.3%, 25.0%, and 33.9%, respectively.

HCUP hospital bed-size categories vary by region and hospital location/teaching status. Small/medium/large cutoffs (number of beds) are: Northeast—rural 1–49/50–99/≥100, urban non-teaching 1–124/125–199/≥200, urban teaching 1–249/250–424/≥425; Midwest—rural 1–29/30–49/≥50, urban non-teaching 1–74/75–174/≥175, urban teaching 1–249/250–374/≥375; South—rural 1–39/40–74/≥75, urban non-teaching 1–99/100–199/≥200, urban teaching 1–249/250–449/≥450; West—rural 1–24/25–44/≥45, urban non-teaching 1–99/100–174/≥175, urban teaching 1–199/200–324/≥325.

### 3.2. In-Hospital Outcomes by Frailty

Adverse in-hospital outcomes increased across HFRS categories (Table 2). In-hospital mortality was 1.2% in the low-HFRS group, 7.1% in the intermediate group, and 9.7% in the high group. Mechanical ventilation occurred in 0.9%, 7.9%, and 16.2%, and acute ischemic stroke in 0.2%, 1.3%, and 6.4%, respectively.

Major bleeding occurred in 1.2% of the low-HFRS group, 4.8% of the intermediate group, and 9.3% of the high group. Circulatory support was uncommon overall but increased from 0.07% in the low-HFRS group to 0.52% in the intermediate group and 0.69% in the high group.

Hospital resource use also differed across HFRS categories. Median length of stay was 3 days (IQR 2–4) in the low-HFRS group, 5 days (IQR 3–7) in the intermediate group, and 7 days (IQR 5–12) in the high group. Mean total hospital charges were approximately USD 39,713, USD 73,009, and USD 116,629, respectively.

### 3.3. Multivariable Analysis

After multivariable adjustment, higher HFRS categories remained associated with each selected clinical outcome.

Compared with the low-HFRS group, the high-HFRS group had higher odds of in-hospital mortality (aOR 8.30, 95% CI 7.24–9.52), mechanical ventilation (aOR 27.74, 95% CI 24.59–31.31), circulatory support (aOR 19.18, 95% CI 11.83–31.11), and acute ischemic stroke (aOR 37.61, 95% CI 30.45–46.46).

High HFRS was also associated with major bleeding (aOR 7.65, 95% CI 6.68–8.76). Intermediate HFRS was associated with mortality (aOR 6.11, 95% CI 5.74–6.51), mechanical ventilation (aOR 11.49, 95% CI 10.70–12.33), circulatory support (aOR 12.17, 95% CI 9.46–15.66), acute ischemic stroke (aOR 6.91, 95% CI 5.93–8.04), and major bleeding (aOR 3.76, 95% CI 3.53–4.00).

**Figure 1:**
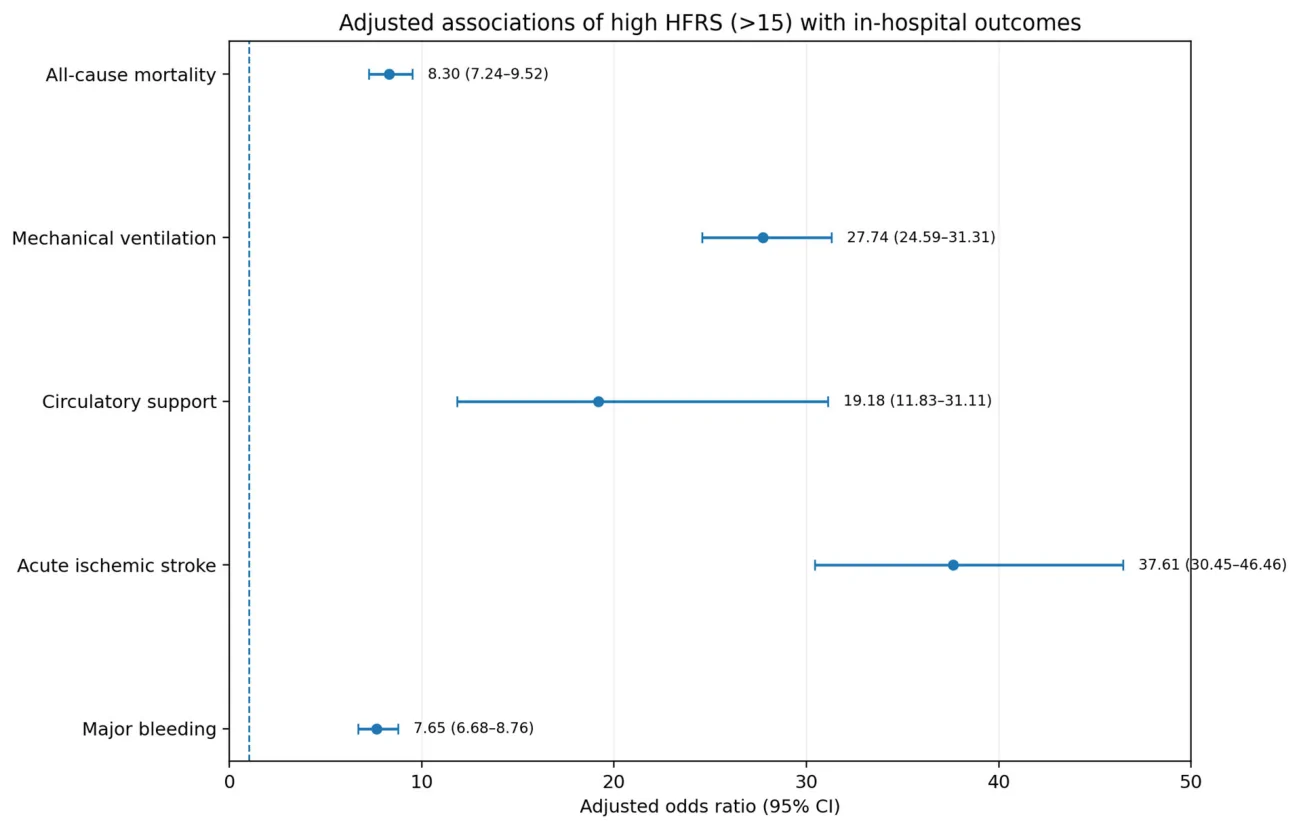
Adjusted odds ratios (95% CIs) for selected in-hospital outcomes comparing high HFRS (>15) with low HFRS (<5). Models account for NIS survey design and the covariates listed in the Methods.

Figure 1 shows these adjusted estimates. The sensitivity analyses are presented below.

### 3.4. Sensitivity Analyses

Sensitivity analyses generally supported an association between HFRS and adverse outcomes, but the magnitude varied by analysis. Among patients aged ≥65 years, high versus low HFRS was associated with mortality (aOR 6.46), mechanical ventilation (aOR 18.97), circulatory support (aOR 14.47), acute ischemic stroke (aOR 28.05), and major bleeding (aOR 5.97). Corresponding estimates among patients aged ≥75 years were 5.18, 15.05, 12.70, 22.56, and 4.74, respectively. Additional adjustment for available PE-severity markers attenuated the high-versus-low association for mortality to aOR 3.76 and for circulatory support to aOR 4.43, while associations with mechanical ventilation (aOR 18.15), acute ischemic stroke (aOR 30.95), and major bleeding (aOR 6.42) remained.

When 19 HFRS categories that could plausibly represent acute conditions during the index admission were removed, only 174 unweighted hospitalizations (0.08% of the weighted cohort) remained in the modified high-HFRS category. We therefore compared modified HFRS ≥5 with <5. Associations were substantially attenuated: mortality aOR 1.30 (95% CI 1.21–1.39), mechanical ventilation aOR 1.79 (95% CI 1.67–1.91), acute ischemic stroke aOR 5.84 (95% CI 5.13–6.65), and major bleeding aOR 2.51 (95% CI 2.34–2.69); circulatory support was not significantly associated (aOR 1.14, 95% CI 0.83–1.56). In an exploratory mortality model, adding HFRS category increased the weighted C-statistic from 0.682 to 0.783 and slightly reduced the weighted Brier score from 0.02993 to 0.02910. Detailed sensitivity results are provided in Supplementary Table S3.

### 3.5. Discharge Disposition by HFRS Category

**Figure 2:**
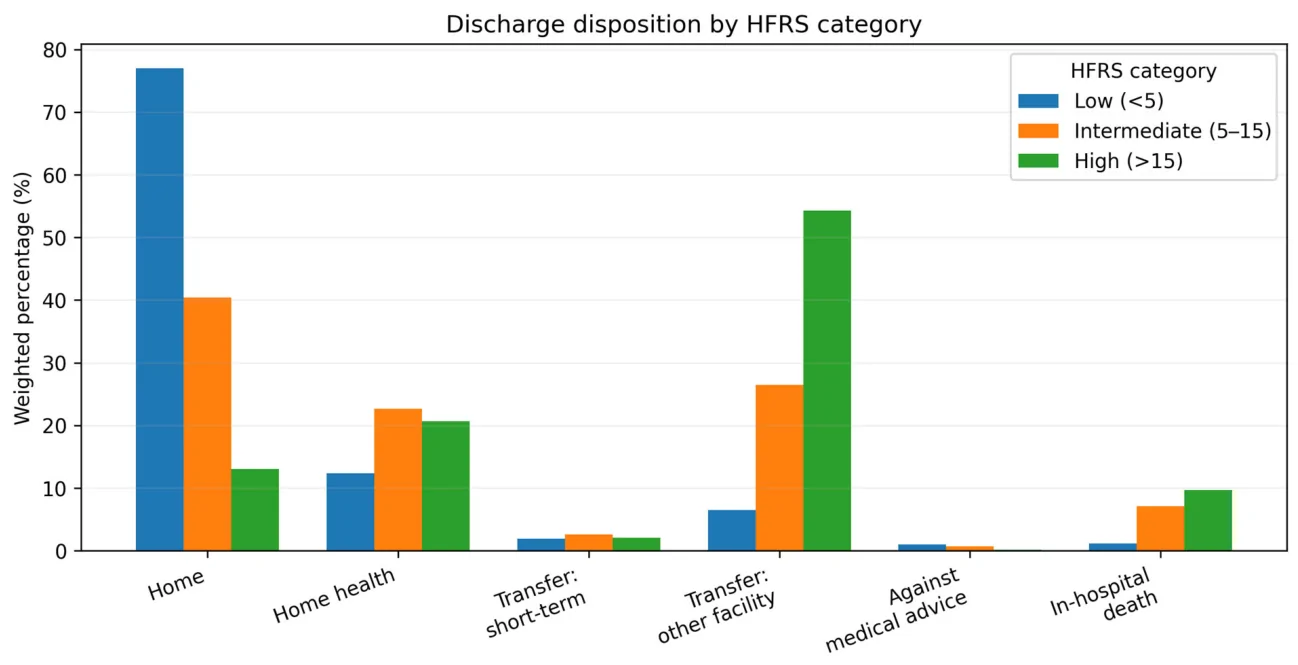
Weighted discharge disposition by HFRS category.

Hospital discharge disposition differed across HFRS categories (Figure 2). Routine discharge home occurred in 77.0% of low-HFRS hospitalizations, 40.4% of intermediate-HFRS hospitalizations, and 13.0% of high-HFRS hospitalizations. Home health care was used in 12.4%, 22.6%, and 20.7%, respectively.

Transfer to another post-acute facility increased from 6.5% in the low-HFRS group to 26.5% in the intermediate group and 54.3% in the high group. Transfer to a short-term hospital was 1.9%, 2.7%, and 2.1%, respectively.

In-hospital death accounted for 1.2%, 7.1%, and 9.7% of discharge dispositions in the low-, intermediate-, and high-HFRS groups, respectively. Discharge against medical advice remained uncommon in all groups.

Overall, higher HFRS was associated with a marked shift away from routine discharge home and toward post-acute institutional care.

## 4. Discussion

In this nationwide analysis of adults hospitalized with acute PE, higher HFRS categories were associated with mortality, mechanical ventilation, circulatory support, ischemic stroke, major bleeding, longer hospital stay, higher hospital charges, and non-routine discharge. HFRS was recalculated so that each three-character category contributed its weight only once per hospitalization, consistent with the original score [13]. This changed the HFRS distribution and reduced several effect estimates, but the overall pattern of association remained.

The sensitivity analyses help clarify these findings. Associations persisted when analyses were restricted to patients aged ≥65 or ≥75 years and when available PE-severity markers were added to the model. In contrast, removing HFRS components that could reflect acute conditions reduced several associations substantially. This suggests that part of the association may result from overlap between HFRS diagnoses and events occurring during the same hospitalization. The primary estimates therefore should not be interpreted as the effect of pre-existing frailty alone.

The strongest associations were observed for mechanical ventilation and acute ischemic stroke. These outcomes also illustrate the limitation of using same-admission diagnosis codes to construct HFRS, because respiratory failure and cerebral infarction are among the HFRS components. The attenuation after excluding acute-prone components suggests that both underlying vulnerability and coding overlap may contribute to the observed associations.

Prior studies have reported worse outcomes among frail patients with PE [15,16]. Our analysis adds a large national sample and a three-level HFRS classification, but it also shows that results are sensitive to how HFRS is constructed in an administrative discharge dataset. The age-restricted analyses support an association in the older population for which HFRS was originally developed, while the acute-component sensitivity analysis argues for caution when using the index admission itself to define frailty.

Discharge disposition also differed across HFRS categories. Patients with higher HFRS were less often discharged home and more often required post-acute care or died in hospital. Discharge against medical advice was uncommon in all groups. These findings suggest that higher HFRS is also associated with greater post-discharge care needs.

These findings do not establish that HFRS should be used to choose thrombolysis, catheter-based treatment, level of monitoring, or other PE management. The exploratory improvement in mortality discrimination is hypothesis-generating only, particularly because HFRS and outcomes are derived from the same hospitalization. Prospective studies comparing HFRS with clinical frailty measures and established PE risk tools are needed before clinical implementation.

This study has several strengths, including a large nationally representative dataset and evaluation of multiple in-hospital outcomes, resource use, and discharge disposition. The HFRS also provides a reproducible method that can be applied to administrative data, although its limitations in this setting must be considered.

## 5. Limitations

Several limitations should be considered. First, the NIS does not provide diagnosis-level present-on-admission indicators. HFRS was therefore based on diagnoses recorded during the index hospitalization, and some components may have developed after admission. The weaker associations after excluding acute-prone components suggest that this limitation affected the estimates. Second, the original HFRS was developed in adults aged ≥75 years and can use diagnoses from prior emergency admissions; the NIS does not allow patient-level longitudinal linkage, so our score used the index admission only. Although age-restricted analyses showed a similar direction of association, the performance of HFRS in younger adults remains uncertain. Third, the NIS lacks vital signs, biomarkers, right-ventricular imaging, and validated PE severity scores. Diagnosis-coded markers such as cardiogenic shock or cardiac arrest also lack reliable timing, so severity-adjusted analyses may include events that occurred after admission. Fourth, frailty and comorbidity are difficult to separate in an administrative score. The extended comorbidity analysis did not remove the associations, but residual confounding remains possible. Fifth, the NIS captures hospitalizations rather than unique patients and includes only in-hospital outcomes. Finally, the exploratory discrimination analysis does not establish calibration, clinical benefit, or added value beyond validated PE risk tools.

## 6. Conclusions

In this national cohort of hospitalizations for acute PE, higher HFRS categories were associated with worse in-hospital outcomes. Associations remained in older patients and after adjustment for available PE-severity markers but were much weaker after excluding HFRS components that may reflect acute conditions. HFRS derived from same-admission administrative data should therefore be interpreted cautiously. Prospective validation is needed before it is used for treatment decisions or as an additional prognostic tool in acute PE.

## Figures and Tables

**Table 1:** Patient and hospital characteristics and diagnosis-coded chronic comorbidities, stratified by Hospital Frailty Risk Score (HFRS) category.

Variable	HFRS < 5	HFRS 5–15	HFRS > 15	*p*-Value
NIS discharge weight (Number, %)	739,520 (67.1%)	346,740 (31.5%)	16,015 (1.5%)	—
Age, years (mean)	60.10	68.97	74.96	<0.001
**Age group categories, %**				<0.001
Under 50 years	25.9	11.0	4.3	
50 to 75 years	55.5	51.1	39.7	
Over 75 years	18.6	37.9	55.9	
Female, %	50.0	55.2	58.4	<0.001
Weekend admission, %	23.2	24.2	25.2	<0.001
**Race/ethnicity, %**				<0.001
White	71.1	71.5	70.2	
Black	19.6	19.7	21.5	
Hispanic	5.9	5.4	5.3	
Asian or Pacific Islander	0.9	1.0	1.1	
Native American	0.4	0.4	0.3	
Other	2.2	2.0	1.6	
**Hospital region, %**				<0.001
Northeast	19.3	17.0	14.0	
Midwest	25.1	26.6	32.3	
South	39.9	39.6	39.7	
West	15.6	16.7	14.1	
**Hospital bed size, %**				<0.001
Small	22.8	20.4	20.0	
Medium	29.7	28.7	28.3	
Large	47.6	50.9	51.7	
**Hospital location/teaching status, %**				<0.001
Rural	9.9	8.6	7.2	
Urban non-teaching	22.1	19.9	16.5	
Urban teaching	68.0	71.5	76.4	
**Median ZIP income, %**				<0.001
1st quartile	28.5	30.0	30.6	
2nd quartile	26.7	26.5	26.2	
3rd quartile	24.7	24.1	23.2	
4th quartile	20.1	19.4	20.0	
**Primary expected payer, %**				<0.001
Medicare	44.6	67.2	79.8	
Medicaid	13.2	9.9	6.0	
Private insurance	34.1	18.1	11.1	
Self-pay	4.9	2.5	1.2	
No charge	0.4	0.2	0.1	
Other	2.9	2.2	1.8	
**Chronic comorbidities, %**				
Heart failure	12.9	27.2	31.9	<0.001
Valvular heart disease	5.3	8.8	11.4	<0.001
Hypertension	57.9	72.2	78.1	<0.001
Hyperlipidemia	34.2	43.0	46.2	<0.001
Atrial fibrillation	9.7	19.2	25.1	<0.001
Current or prior smoking	37.7	44.0	39.1	<0.001
Dementia	1.5	13.9	39.2	<0.001
Chronic kidney disease	7.3	25.0	33.9	<0.001
Chronic lung disease	22.9	31.3	28.4	<0.001
Obesity	27.9	24.3	18.5	<0.001
Anemia	18.2	35.0	44.9	<0.001
Thrombocytopenia	4.4	8.2	10.4	<0.001
Coagulopathy	6.3	7.3	8.1	<0.001
Peripheral vascular disease	2.6	4.7	6.0	<0.001
Prior myocardial infarction	4.5	6.2	6.6	<0.001
Malignancy	14.1	18.6	13.9	<0.001

HFRS, Hospital Frailty Risk Score. Values are weighted percentages unless otherwise indicated. *p* values represent overall comparisons across the three HFRS categories.

**Table 2:** In-hospital outcomes and resource use, stratified by Hospital Frailty Risk Score (HFRS) category.

Variable	HFRS < 5	HFRS 5–15	HFRS > 15	*p*-Value
NIS discharge weight (Number, %)	739,520 (67.1%)	346,740 (31.5%)	16,015 (1.5%)	—
All-cause mortality	1.2	7.1	9.7	<0.001
Mechanical ventilation	0.9	7.9	16.2	<0.001
Circulatory support	0.07	0.52	0.69	<0.001
Acute ischemic stroke	0.2	1.3	6.4	<0.001
Major bleeding	1.2	4.8	9.3	<0.001
Intracranial hemorrhage	0.2	0.9	2.8	<0.001
Retroperitoneal bleeding	0.09	0.45	0.87	<0.001
Gastrointestinal bleeding	0.9	3.5	6.0	<0.001
Length of stay, days, median (IQR)	3 (2–4)	5 (3–7)	7 (5–12)	<0.001
Total hospital charges, USD (mean)	39,713	73,009	116,629	<0.001

HFRS, Hospital Frailty Risk Score. Values are weighted percentages unless otherwise indicated. *p* values represent overall comparisons across the three HFRS categories.

## Data Availability

The National Inpatient Sample is available from the Healthcare Cost and Utilization Project (HCUP) Central Distributor to eligible users who complete the required Data Use Agreement training and execute the HCUP Data Use Agreement. Under the HCUP Data Use Agreement, the authors are not permitted to redistribute the raw NIS data. Information on obtaining the NIS is available at https://www.hcup-us.ahrq.gov/ (accessed on 10 August 2026).

## Supplementary Materials

The following supporting information can be downloaded at https://www.mdpi.com/article/10.3390/jcm15208107/s1. Table S1: ICD-10 categories and weights used to calculate the Hospital Frailty Risk Score; Table S2: Weighted prevalence of HFRS components by HFRS category; Table S3: Sensitivity analyses for selected outcomes; Table S4: Outcome definitions and coding approach.
