# Supplementary material for: Frailty Impact on In-Hospital Outcomes Among Patients Hospitalized with Acute Pulmonary Embolism: A National Inpatient Sample Analysis
Source: J Clin Med. 2026 Oct 19;15(20):8107. doi: 10.3390/jcm15208107 (PMC13706008; doi:10.3390/jcm15208107)
Supplement: Supplementary file 1 [file jcm-15-08107-s001.zip › jcm-4532197-supplementary.pdf]

Supplementary Materials

Frailty Impact on In-Hospital Outcomes Among Patients Hospitalized with Acute Pulmonary Embolism: A National Inpatient Sample Analysis

Table S1. ICD-10 categories and weights used to calculate the Hospital Frailty Risk Score

ICD-10 category	Description	Weight
F00	Dementia in Alzheimer	7.1
G81	Hemiplegia	4.4
G30	Alzheimer's disease	4.0
I69	Sequelae of cerebrovascular disease (secondary codes)	3.7
R29	Other symptoms and signs involving the nervous and musculoskeletal systems (R29.6 Tendency to fall)	3.6
N39	Other disorders of urinary system (includes UTI and urinary incontinence)	3.2
F05	Delirium (non-substance induced)	3.2
W19	Unspecified fall	3.2
S00	Superficial injury of head	3.2
R31	Unspecified hematuria	3.0
B96	Other bacterial agents (secondary code)	2.9
R41	Cognitive function and awareness symptoms	2.7
R26	Abnormalities of gait and mobility	2.6
I67	Other cerebrovascular diseases	2.6
R56	Convulsions	2.6
R40	Somnolence, stupor and coma	2.5
T83	GU prosthetic device complications	2.4
S06	Intracranial injury	2.4
S42	Fracture of shoulder and upper arm	2.3
E87	Fluid, electrolyte, acid-base disorders	2.3
M25	Other joint disorders	2.3
E86	Volume depletion	2.3
R54	Senility	2.2
Z50	Care involving use of rehabilitation procedures	2.1
F03	Unspecified dementia	2.1
W18	Other fall on same level	2.1
Z75	Problems related to medical facilities	2.0
F01	Vascular dementia	2.0
S80	Superficial injury of lower leg	2.0
L03	Cellulitis	2.0
H54	Blindness and low vision	1.9
E53	Vitamin B deficiency	1.9
G20	Parkinson's disease	1.8
R55	Syncope and collapse	1.8
S22	Fracture of ribs/sternum/thoracic spine	1.8
K59	Functional intestinal disorders	1.8
N17	Acute renal failure	1.8
L89	Pressure ulcer	1.7
Z22	Carrier of infectious disease	1.7
B95	Streptococcus/staphylococcus (secondary code)	1.7
L97	Ulcer of lower limb	1.6
R44	Sensory perception symptoms	1.6
K26	Duodenal ulcer	1.6
I95	Hypotension	1.6

ICD-10 category	Description	Weight
N19	Unspecified renal failure	1.6
A41	Septicemia	1.6
X59	Exposure to unspecified factor	1.5
Z87	Personal history of disease	1.5
J96	Respiratory failure	1.5
M19	Arthrosis	1.5
G40	Epilepsy	1.5
M81	Osteoporosis without fracture	1.4
S72	Fracture of femur	1.4
S32	Lumbar spine and pelvis fracture	1.4
E16	Pancreatic internal secretion disorders	1.4
R94	Abnormal function study results	1.4
N18	Chronic renal failure	1.4
R33	Urinary retention	1.3
R69	Unspecified morbidity	1.3
N28	Kidney/ureter disorders	1.3
G31	Degenerative nervous system diseases	1.2
R32	Urinary incontinence	1.2
S09	Other head injuries	1.2
R45	Emotional state symptoms	1.2
G45	Transient ischemic attack	1.2
Z74	Care-provider dependency	1.1
M79	Soft tissue disorders	1.1
W06	Fall from bed	1.1
S01	Open wound of head	1.1
A09	Infectious diarrhea/gastroenteritis	1.1
J18	Pneumonia (unspecified organism)	1.1
A04	Other bacterial intestinal infections	1.1
R02	Gangrene	1.0
J69	Aspiration pneumonitis	1.0
R47	Speech disturbance	1.0
E55	Vitamin D deficiency	1.0
Z93	Artificial opening status	1.0
R63	Food/fluid intake symptoms	0.9
H91	Hearing loss	0.9
W10	Fall from stairs/steps	0.9
W01	Slip/trip fall	0.9
E05	Hyperthyroidism	0.9
M41	Scoliosis	0.9
R13	Dysphagia	0.8
Z99	Dependence on devices	0.8
M80	Osteoporosis with fracture	0.8
K92	Other digestive diseases	0.8
I63	Cerebral infarction	0.8
N20	Kidney/ureter stones	0.7
F10	Alcohol-related disorders	0.7
Y84	Medical procedure complication	0.7
R00	Abnormal heartbeat	0.7
J22	Acute lower respiratory infection	0.7
Z73	Life-management difficulty	0.6
Z91	Personal risk factors history	0.5
S51	Open wound of forearm	0.5
F32	Depressive episode	0.5
M48	Spinal stenosis	0.5
E83	Mineral metabolism disorder	0.4
M15	Polyarthrosis	0.4

ICD-10 category	Description	Weight
D64	Anemia	0.4
L08	Local skin infection	0.4
R11	Nausea and vomiting	0.3
K52	Noninfective gastroenteritis	0.3
R50	Fever of unknown origin	0.1
Z60	Social environment problems	1.8
R79	Abnormal blood chemistry	0.6
U80	Antibiotic resistance (penicillin)	0.8
Y95	Nosocomial condition	1.2

Table S2. Weighted prevalence of HFRS components by HFRS category

ICD-10 category	Description	Weight	HFRS <5, %	HFRS 5–15, %	HFRS >15, %
F00	Dementia in Alzheimer	7.1	NR	NR	NR
G81	Hemiplegia	4.4	0.01	1.00	7.02
G30	Alzheimer's disease	4.0	0.14	3.54	12.80
I69	Sequelae of cerebrovascular disease (secondary codes)	3.7	0.27	5.14	17.33
R29	Other symptoms and signs involving the nervous and musculoskeletal systems (R29.6 Tendency to fall)	3.6	0.07	2.97	17.98
N39	Other disorders of urinary system (includes UTI and urinary incontinence)	3.2	0.91	14.35	52.48
F05	Delirium (non-substance induced)	3.2	0.02	1.44	9.65
W19	Unspecified fall	3.2	0.15	2.89	12.86
S00	Superficial injury of head	3.2	0.03	0.90	4.93
R31	Unspecified hematuria	3.0	0.43	3.68	11.99
B96	Other bacterial agents (secondary code)	2.9	0.17	5.55	30.85
R41	Cognitive function and awareness symptoms	2.7	0.18	2.38	8.90
R26	Abnormalities of gait and mobility	2.6	0.19	1.94	6.56
I67	Other cerebrovascular diseases	2.6	0.09	0.62	2.59
R56	Convulsions	2.6	0.19	1.33	4.12
R40	Somnolence, stupor and coma	2.5	0.20	1.82	7.74
T83	GU prosthetic device complications	2.4	0.01	0.65	6.03
S06	Intracranial injury	2.4	0.04	0.51	2.19
S42	Fracture of shoulder and upper arm	2.3	0.08	0.64	2.03
E87	Fluid, electrolyte, acid-base disorders	2.3	10.03	48.53	68.40
M25	Other joint disorders	2.3	0.80	2.93	5.18
E86	Volume depletion	2.3	1.11	11.81	30.03
R54	Senility	2.2	0.04	0.49	1.94
Z50	Care involving use of	2.1	NR	NR	NR

ICD-10 category	Description	Weight	HFRS <5, %	HFRS 5–15, %	HFRS >15, %
	rehabilitation procedures				
F03	Unspecified dementia	2.1	1.19	8.90	22.23
W18	Other fall on same level	2.1	0.12	1.63	5.31
Z75	Problems related to medical facilities	2.0	0.02	0.18	0.41
F01	Vascular dementia	2.0	0.08	0.87	3.87
S80	Superficial injury of lower leg	2.0	0.05	0.46	2.06
L03	Cellulitis	2.0	0.63	2.67	4.46
H54	Blindness and low vision	1.9	0.33	1.59	2.97
E53	Vitamin B deficiency	1.9	0.46	1.72	3.59
G20	Parkinson's disease	1.8	0.50	2.26	5.68
R55	Syncope and collapse	1.8	1.34	4.27	7.52
S22	Fracture of ribs/sternum/thoracic spine	1.8	0.28	1.81	5.43
K59	Functional intestinal disorders	1.8	3.13	11.57	18.33
N17	Acute renal failure	1.8	5.46	31.14	51.76
L89	Pressure ulcer	1.7	0.36	3.93	14.27
Z22	Carrier of infectious disease	1.7	0.07	0.37	0.50
B95	Streptococcus/staphylococcus (secondary code)	1.7	0.24	1.99	8.68
L97	Ulcer of lower limb	1.6	0.36	1.49	2.93
R44	Sensory perception symptoms	1.6	0.04	0.29	1.22
K26	Duodenal ulcer	1.6	0.10	0.56	0.84
I95	Hypotension	1.6	2.31	12.59	25.13
N19	Unspecified renal failure	1.6	0.03	0.10	NR
A41	Septicemia	1.6	0.20	3.40	11.52
X59	Exposure to unspecified factor	1.5	NR	NR	NR
Z87	Personal history of disease	1.5	23.21	38.36	38.96
J96	Respiratory failure	1.5	17.70	48.53	61.07
M19	Arthrosis	1.5	5.33	12.55	16.02
G40	Epilepsy	1.5	1.53	4.71	9.18
M81	Osteoporosis without fracture	1.4	1.45	5.19	8.74
S72	Fracture of femur	1.4	0.12	0.66	2.50
S32	Lumbar spine and pelvis fracture	1.4	0.12	0.61	2.12
E16	Pancreatic internal secretion disorders	1.4	0.06	0.51	1.69

ICD-10 category	Description	Weight	HFRS <5, %	HFRS 5–15, %	HFRS >15, %
R94	Abnormal function study results	1.4	0.67	1.35	2.28
N18	Chronic renal failure	1.4	7.34	24.96	33.94
R33	Urinary retention	1.3	0.60	4.00	12.24
R69	Unspecified morbidity	1.3	NR	NR	NR
N28	Kidney/ureter disorders	1.3	1.38	3.35	5.06
G31	Degenerative nervous system diseases	1.2	0.19	0.84	2.75
R32	Urinary incontinence	1.2	0.31	1.68	4.78
S09	Other head injuries	1.2	0.03	0.32	1.09
R45	Emotional state symptoms	1.2	0.23	0.96	2.59
G45	Transient ischemic attack	1.2	0.08	0.29	0.84
Z74	Care-provider dependency	1.1	0.37	2.24	7.24
M79	Soft tissue disorders	1.1	2.85	4.86	5.09
W06	Fall from bed	1.1	0.02	0.20	1.09
S01	Open wound of head	1.1	0.08	0.70	2.25
A09	Infectious diarrhea/gastroenteritis	1.1	0.02	0.06	NR
J18	Pneumonia (unspecified organism)	1.1	6.93	14.73	17.73
A04	Other bacterial intestinal infections	1.1	0.21	1.03	2.75
R02	Gangrene	1.0	NR	NR	NR
J69	Aspiration pneumonitis	1.0	0.27	2.90	9.80
R47	Speech disturbance	1.0	0.17	1.32	6.77
E55	Vitamin D deficiency	1.0	1.20	2.89	4.90
Z93	Artificial opening status	1.0	0.88	2.62	4.46
R63	Food/fluid intake symptoms	0.9	0.81	2.09	3.68
H91	Hearing loss	0.9	0.91	2.93	5.00
W10	Fall from stairs/steps	0.9	0.04	0.17	NR
W01	Slip/trip fall	0.9	0.11	0.60	1.59
E05	Hyperthyroidism	0.9	0.41	0.77	1.00
M41	Scoliosis	0.9	0.29	0.70	1.03
R13	Dysphagia	0.8	0.84	4.65	16.83
Z99	Dependence on devices	0.8	3.56	11.42	12.58
M80	Osteoporosis with fracture	0.8	0.05	0.23	0.47
K92	Other digestive diseases	0.8	0.75	2.87	5.34
I63	Cerebral infarction	0.8	0.18	1.26	6.37
N20	Kidney/ureter stones	0.7	0.53	1.12	1.75

ICD-10 category	Description	Weight	HFRS <5, %	HFRS 5–15, %	HFRS >15, %
F10	Alcohol-related disorders	0.7	3.09	4.72	4.50
Y84	Medical procedure complication	0.7	0.25	1.12	3.59
R00	Abnormal heartbeat	0.7	5.46	8.43	12.05
J22	Acute lower respiratory infection	0.7	0.01	0.02	NR
Z73	Life-management difficulty	0.6	0.02	0.02	NR
Z91	Personal risk factors history	0.5	8.49	12.62	14.80
S51	Open wound of forearm	0.5	0.01	0.09	0.47
F32	Depressive episode	0.5	10.54	15.98	18.26
M48	Spinal stenosis	0.5	1.13	2.80	4.68
E83	Mineral metabolism disorder	0.4	3.02	10.47	15.80
M15	Polyarthrosis	0.4	0.21	0.37	0.53
D64	Anemia	0.4	7.61	14.93	19.39
L08	Local skin infection	0.4	0.04	0.11	NR
R11	Nausea and vomiting	0.3	0.92	1.71	1.59
K52	Noninfective gastroenteritis	0.3	0.60	1.26	1.50
R50	Fever of unknown origin	0.1	0.79	1.01	1.25
Z60	Social environment problems	1.8	0.11	0.41	0.78
R79	Abnormal blood chemistry	0.6	3.08	5.20	6.59
U80	Antibiotic resistance (penicillin)	0.8	NR	NR	NR
Y95	Nosocomial condition	1.2	0.18	1.46	2.90

Percentages are weighted within each HFRS category. Each three-character HFRS category was counted at most once per hospitalization. NR, not reported because the underlying unweighted cell count was ≤10, in accordance with HCUP reporting requirements.

Table S3. Sensitivity analyses for selected outcomes

Analysis	Outcome	Comparison	aOR	95% CI	P value
Primary analysis	All-cause mortality	High vs low	8.30	7.24–9.52	<0.001
Primary analysis	Mechanical ventilation	High vs low	27.74	24.59–31.31	<0.001
Primary analysis	Circulatory support	High vs low	19.18	11.83–31.11	<0.001
Primary analysis	Acute ischemic stroke	High vs low	37.61	30.45–46.46	<0.001
Primary analysis	Major bleeding	High vs low	7.65	6.68–8.76	<0.001
Age ≥65 years	All-cause mortality	High vs low	6.46	5.54–7.54	<0.001
Age ≥65 years	Mechanical ventilation	High vs low	18.97	16.36–22.00	<0.001
Age ≥65 years	Circulatory support	High vs low	14.47	7.13–29.38	<0.001
Age ≥65 years	Acute ischemic stroke	High vs low	28.05	21.93–35.88	<0.001
Age ≥65 years	Major bleeding	High vs low	5.97	5.07–7.03	<0.001
Age ≥75 years	All-cause mortality	High vs low	5.18	4.31–6.23	<0.001
Age ≥75 years	Mechanical ventilation	High vs low	15.05	12.29–18.43	<0.001
Age ≥75 years	Circulatory support	High vs low	12.70	3.49–46.20	<0.001
Age ≥75 years	Acute ischemic stroke	High vs low	22.56	16.30–31.21	<0.001
Age ≥75 years	Major bleeding	High vs low	4.74	3.85–5.82	<0.001
Additional adjustment for available PE severity markers	All-cause mortality	High vs low	3.76	3.07–4.61	<0.001
Additional adjustment for available PE severity markers	Mechanical ventilation	High vs low	18.15	15.55–21.19	<0.001
Additional adjustment for available PE severity markers	Circulatory support	High vs low	4.43	2.57–7.65	<0.001
Additional adjustment for available PE severity markers	Acute ischemic stroke	High vs low	30.95	24.96–38.36	<0.001
Additional adjustment for available PE severity markers	Major bleeding	High vs low	6.42	5.60–7.37	<0.001
Extended diagnosis-coded comorbidity adjustment	All-cause mortality	High vs low	8.45	7.33–9.74	<0.001
Extended diagnosis-coded comorbidity adjustment	Mechanical ventilation	High vs low	28.16	24.85–31.90	<0.001
Extended diagnosis-coded comorbidity	Circulatory support	High vs low	10.50	6.27–17.56	<0.001

Analysis	Outcome	Comparison	aOR	95% CI	P value
adjustment					
Extended diagnosis-coded comorbidity adjustment	Acute ischemic stroke	High vs low	40.79	32.75–50.80	<0.001
Extended diagnosis-coded comorbidity adjustment	Major bleeding	High vs low	5.74	4.96–6.65	<0.001
Acute-prone HFRS components excluded	All-cause mortality	HFRS ≥5 vs <5	1.30	1.21–1.39	<0.001
Acute-prone HFRS components excluded	Mechanical ventilation	HFRS ≥5 vs <5	1.79	1.67–1.91	<0.001
Acute-prone HFRS components excluded	Circulatory support	HFRS ≥5 vs <5	1.14	0.83–1.56	0.430
Acute-prone HFRS components excluded	Acute ischemic stroke	HFRS ≥5 vs <5	5.84	5.13–6.65	<0.001
Acute-prone HFRS components excluded	Major bleeding	HFRS ≥5 vs <5	2.51	2.34–2.69	<0.001

The acute-component sensitivity analysis excluded N39, F05, B96, R56, R40, S06, E87, E86, R55, N17, B95, A41, I95, J96, I63, J18, J22, Y95, and R50 from HFRS. Because only 174 unweighted hospitalizations remained in the modified >15 category, this analysis compared HFRS ≥5 with <5. The PE-severity sensitivity model included saddle PE, cardiogenic shock, cardiac arrest, ventricular tachycardia, and ventricular fibrillation. The extended comorbidity model additionally included diabetes mellitus, dementia, chronic kidney disease, hypertension, heart failure, atrial fibrillation, chronic lung disease, anemia, thrombocytopenia, coagulopathy, peripheral vascular disease, and chronic liver disease.

Exploratory mortality discrimination analysis:

Model	Weighted C-statistic	Weighted Brier score
Base covariates	0.682	0.02993
Base + HFRS category	0.783	0.02910

Adding HFRS to the mortality model increased the weighted C-statistic from 0.682 to 0.783 and reduced the weighted Brier score from 0.02993 to 0.02910. This was an exploratory in-sample analysis. We did not evaluate formal calibration, net reclassification, or clinical net benefit.

Table S4. Outcome definitions and coding approach

Outcome	Operational definition	ICD-10-CM/PCS coding approach in analytic dataset
All-cause in-hospital mortality	Death during index hospitalization	NIS DIED variable.
Mechanical ventilation	Invasive respiratory ventilation during the hospitalization	ICD-10-PCS 5A1935Z, 5A1945Z, 5A1955Z, or 5A19054 in any procedure field.
Circulatory support	Mechanical circulatory support during the hospitalization	Any ICD-10-PCS code beginning 5A1522, 5A0221, 5A0211, or 02HA3R.
Acute ischemic stroke	Acute cerebral infarction during the hospitalization	Any ICD-10-CM code in I63.*.
Intracranial hemorrhage	Nontraumatic intracranial hemorrhage	Any ICD-10-CM code in I60.*, I61.*, or I62.*.
Gastrointestinal bleeding	Diagnosis-coded gastrointestinal hemorrhage	ICD-10-CM K92.0, K92.1, K92.2; K25.0/K25.2/K25.4/K25.6; K26.0/K26.2/K26.4/K26.6; K27.0/K27.2/K27.6; K28.0/K28.2/K28.4/K28.6; or K29.01/K29.11/K29.21/K29.31/K29.41/K29.51/K29.61/K29.71/K29.81/K29.91.
Retroperitoneal bleeding	Retroperitoneal hemorrhage	ICD-10-CM K66.1.
Major bleeding	Composite bleeding outcome	Logical union of intracranial hemorrhage, gastrointestinal bleeding, and retroperitoneal bleeding as defined above.
Length of stay	Days from admission to discharge	NIS LOS variable.
Total hospital charges	Total billed hospital charges for the index hospitalization	NIS TOTCHG variable; no cost-to-charge conversion was applied.
Discharge disposition	Disposition at hospital discharge	NIS DISPUNIF variable.

MACCE, systemic thrombolysis, catheter-directed therapy, and procedure-related bleeding were removed from the revised analysis because their original derivation could not be reconstructed with enough confidence. All retained diagnosis- and procedure-based outcomes were rebuilt from the raw ICD-10-CM/PCS fields using the definitions below.

The code sets in Table S4 were checked against the raw diagnosis and procedure fields used in the final analysis.
